# 

**Published:** 1850-03

**Authors:** 


### CONTENTS

### OF NO. III.

### ORIGINAL COMMUNICATIONS.

### ESSAYS AND CASES.

ART. I.—The Diagnostic Value of the Urine. A Paper read before a meeting of the Montgomery Medical Society, held at Clarksville, Tenn., Nov. 3, 1849. By E. B. HASKINS, M. D. - - - 185

ART. II.—The History, Diagnosis, and Treatment of Edematous Laryngitis. By ELISHA BARTLETT, M. D., Professor of the Theory and Practice of Medicine in the University of Louisville, - - - - 209

ART. III.—Cases at the Louisville Marine Hospital. By DAVID W. YANDELL, M. D., of Louisville, Ky., Attending Physician for the Faculty at the Hospital, - 241

ART. IV.—Programme of a Premium offered by the Society of Pharmacy, of Paris. Translated for this Journal from the Journal of Pharmacy, December, 1849. 248

### REVIEWS.

ART. V.—The American Journal of Science and Arts. By Prof. B. SILLIMAN, L. L. D., M. D., &c., &c.; Prof. B. SILLIMAN, Jr., A. M., M. D., &c., and JAMES D. DANA, A. M. New Haven, Conn. - - - 252

ART. VI.—Braithwaite's Retrospect of Practical Medicine and Surgery. Part 20th.

The Half-Yearly Abstract of the Medical Sciences: Being a practical and analytical digest of the contents of the British and continental medical works, published during the preceding six months. Edited by W. H. RANKING, M. D., Cantab., &c. - - - - - 257

SELECTIONS FROM AMERICAN AND FOREIGN JOURNALS.

Typhus Fever, - - - - - - - 258
Treatment of Diarrhea and Dysentery of Fever, - - 258
Employment of Manganese in Anemic and other affections, - 259
Treatment of Nevus by a Solution of Iodine, - - 262
On the Extraction of Foreign Bodies from the Esophagus, - 262
On Treatment of Fissures of the Anus, - - - 264
Salivation Treated by Nitrate of Silver, - - - 266
On the Treatment of External Hemorrhoids by Potassa Fusa, - 268

ORIGINAL INTELLIGENCE.

The Louisville Medical School, - - - - 269
Colleges in the United States, - - - - - 270
American Medical Association, - - - - 273
The Cause of Cholera, - - - - - - 275
Tilden & Co.'s Alcoholic Extracts, - - - - 275
Prof. Bartlett's Essay on Edematous Laryngitis, - - 276
Obituary, - - - - - - - - 276

